# TPLE: A Reliable Data Delivery Scheme for On-Road WSN Traffic Monitoring

**DOI:** 10.3390/s17010044

**Published:** 2016-12-27

**Authors:** Rui Wang, Fei Chang, Suli Ren

**Affiliations:** 1 School of Computer and Communication Engineering, University of Science and Technology Beijing, Beijing 100083, China; changfeifei@outlook.com (F.C.); rensuli@126.com (S.R.); 2 Beijing Key Laboratory of Knowledge Engineering for Materials Science, Beijing 100083, China

**Keywords:** intelligent transportation systems, reliable data delivery, wireless sensor networks

## Abstract

In an on-road environment, motor-engines severely disturb the wireless link of a sensor node, leading to high package loss rate, high delivery delay, and poor radio communication quality. The existing data delivery mechanisms, such as the ACK-based retransmission mechanism and window-based link quality estimation mechanism, could not handle these challenges well. To solve this challenge, we propose a Target-Prediction-based Link quality Estimation scheme (TPLE) to realize high quality data delivery in an on-road environment. To perform on-road link quality estimation, TPLE dynamically calculates the track of a nearby vehicle target and estimates target impact on wireless link. Based on the local estimation of link quality, TPLE schedules radio communication tasks effectively. Simulations indicate that our proposed TPLE scheme produces a 94% data delivery rate, its average retransmission number is around 0.8. Our conducted on-road data delivery experiments also indicated a similar result as the computer simulation.

## 1. Introduction

Traffic monitoring is an important part of modern city administration. Real-time road traffic data helps improve transportation efficiency [1], such as road information broadcast and real time weather publication.

Due to features such as low cost, flexible deployment, and easy maintenance, Wireless Sensor Networks are very suitable for establishment of extensive Intelligent Transportation Systems [2]. Some studies [2,3,4,5] have been conducted to collect on-road data by WSNs.

To realize large scale data collection by WSN upon on-road monitoring, there are still many challenges. One challenge is the reliability of data transmission from a sensor node to its destination in an on-road transportation environment. In an on-road traffic environment, the engine and other noises are much stronger as compared to other environments, especially the noise caused by engines. So the reliable wireless data delivery in on-road environments should counter for these obstacles.

We conducted an experiment to point out the aforementioned communication complexity. In that experiment, we used two sensor nodes equipped with CC1000 [6], one as a sender and one as a receiver. We set the distance between two sensor nodes to be 6 m and set the radio power to be 0 dB/m. We conducted the same experiment in three different environments:

(1) Two nodes placed an indoor environment.
(2) Two sensor nodes placed an outdoor non-traffic environment.
(3) Two sensor nodes placed along the road sides (where heavy vehicles run on the road).

**Figure 1:**
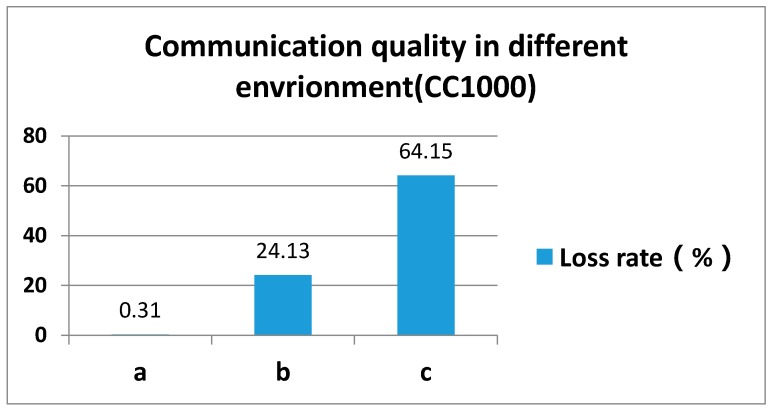
Radio loss rate in different settings.

Each experiment lasted 10 min duration. As shown in Figure 1, the meaning of *y* axis is package loss rate in three different environments. We define 10 percentage points of its value as a span. You can see the package loss rate in environment (**a**) is the minimum, and the maximum packet loss rate achieved was 64.15% where heavy vehicles run on the road. Figure 1 shows the overall package loss rate. In an on-road context, the radio communication quality in non-traffic outdoor environment is better than in the traffic road. The only difference between the two scenarios is the busy vehicle targets running in the road.

From Figure 1 we made the following observations:

(1) The wireless communication quality in on-road environments is relatively poor compared to other environments. The main reason for the worst radio communication quality is engine noises nearby the wireless vicinity.
(2) The running and passing vehicles caused huge distortion to wireless link quality. The unstable and unpredictable nature of link quality gave rise to poor performance of data delivery, such as low delivery rate, high time delay, etc.

Traditionally, in wireless communication, link quality estimation is adopted to support the realization of reliable and low-delay data transmission. We argue that in an on-road traffic environment, the link quality cannot be described by existing link models. Apart from the normal factors that influence link quality, such as background noises, etc., when an on-road traffic environment is referred, the running vehicles cause disruption to wireless links, especially the radio link used in wireless sensor networks. From Figure 1, by comparing scenarios (**b**) and (**c**), intuitively, almost 40% of sent Packets are lost due to the noise of motor engines.

In this paper, we focus on the reliability of on-road data delivery, we have proposed and implemented a reliable data transmission scheme, called TPLE, which can conduct node data delivery according to real time traffic position around the sensor node. Our proposed scheme can effectively estimate the link quality and provide good communication quality. Our contributions are as follows:

1. We proposed the TPLE scheme which can effectively deal with strong noises caused by on-road motor engines targets.
2. With this scheme, we obtain relatively high radio communication quality with the acceptable cost of time delay with respect to data delivery.
3. We developed a real-time application to verify our proposed scheme; our system can be applied in pervasive on-road data collection.

## 2. Related Work

In this part, we briefly introduce the research of applying WSN in on-road monitoring and the research of reliable data delivery mechanisms. 

Coleri et al. [2] proposed the idea of adopting WSN in on-road monitoring. The author proposed a single-magnetic-sensor-based solution for on-road vehicle identification. Also, a two-sensor-based on-the-spot vehicle speed estimation scheme was proposed. In [3], the author proposed a data collection scheme to detect vehicle on a grass. Wireless radio delivers both raw measurement and in-network control information. Radio communication mainly occurs around those sensor nodes near the moving vehicle.

In the existing research of WSN 802.11 networking, the challenges to building reliable data delivery are packet losses and errors. To handle these two challenges, solutions can be classified into two categories:

### 2.1. Frame-Based Solutions

In [4], the author proposed a solution for traffic lights management. Communications occurred among on-vehicle sensors and roadside relays and the solution is that they needed continuous RSSI (Received Signal Strength Indication) sampling, which is energy inefficient.

In [5], the author proposed a detection and classification solution for motor vehicles. The vehicle classification is accomplished by processing data from multiple sensor nodes. A dynamical central node receives radio messages from correlated neighbors and performs the collaborative detection or classification tasks.

All of the aforementioned research work by default assumed a reliable wireless data delivery. However, as we have mentioned earlier, the reliability of data delivery in terms of an on-road environment cannot be omitted to build a practical data collection system. The on-road environment presents new challenges to wireless link estimation research.

### 2.2. Physical-Based Solutions

COLLIE [7] detects collisions from weak signal for 802.11 networks by identifying error patterns within a physical-layer symbol. Moreover, COLLIE adopts a feedback of error packets from the receiver side. COLLIE is not applicable in the WSN field because this mechanism cost is too high with respect to measurement overheads and energy consumption.

SoftRate [8] uses SoftPHY hints to estimate sudden changes in the BER, so that collisions and packet losses can be detected. SoftRate needs access to a physical layer which is difficult to realize in WSN.

Present research on the chip error patterns on IEEE 802.15.4 standards shows that the packet losses can be estimated [9]. Access to a physical layer is also essential here and makes this solution impractical in WSN.

Carried out research about packet loss estimation prediction in WSN with a joint RSSI-LQI based classifier [10], the received packet can be classified into four categories: lost, successfully received, error by collision, and error by weak signal. Then corresponding actions can be adopted to improve the reliability of the data delivery. The disadvantage of this scheme is solutions, [11,12,13] use RTS/CTS to assure a reliable data communication in 802.11 networks. The RTS/CTS overhead is relatively high and it is always disabled in WSN design.

In summary, the existing research in 802.11 networks on how to predict packet loss to realize a reliable data delivery cannot be applied to WSN. There are some studies, such as [10], trying to realize reliable data delivery. However, as we have demonstrated in Figure 1, the noise and disturbance in an on-road context is much higher than typical WSN deployment sites, such as in indoor [14], open field [15], and outdoor [16,17] contexts. In this paper, we present our research on how to tackle the unprecedented scale of disruption in an on-road WSN communication, and we also present our implementation of Target-Prediction-based on a Link quality Estimation system which neither needed any overheads in the physical layer nor used RTS/CTS.

## 3. TPLE: Target-Prediction-Based Link Quality Estimation Mechanism

**Figure 2:**
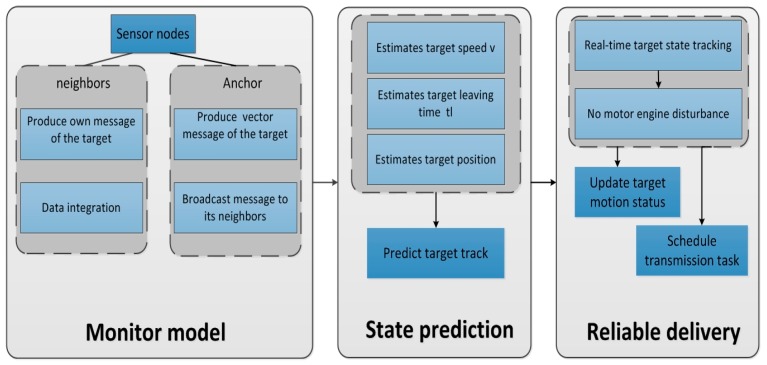
The framework of TPLE.

In this section we propose a mechanism, namely TPLE for on-road data collection systems to reliably deliver data. We firstly describe the ideas of TPLE, and then we describe the TPLE data collection procedures for data collection regarding on-road traffic. The framework of TPLE is shown in Figure 2 as follows.

As shown in Figure 2, TPLE includes three parts: monitor model, state prediction, and reliable delivery. The target can be sensed by sensor nodes when it comes, and the vector message about the incoming target which is produced by Anchor and its neighbors will be integrated to predict the target state. According to real time motion state tracking, the state of the motor engine which causes disturbance to data delivery can be calculated dynamically locally. When the engine is out of the influential scope of a sensor, the sensor will update the target motion status and schedule the transmission task effectively. Thus, a reliable data delivery mechanism is built.

In detail, when the target is sensed by the Anchor, it can produce a message of feature vector I, which includes the message of the time instant that the target passes the Anchor and the signal strength of monitoring target when the target reaches the Anchor. Then, the Anchor will broadcast the feature vector I to its next neighbor node. When the next neighbor received the I, in a short time, it can also produce a feature vector about the target, and will broadcast to its next neighbor node. We can estimate the target speed and calculate the leaving time when the target is out of the influential scope of each sensor by using such a form for message transmission of the feature vector. So, the sensor can reschedule the transmission tasks according to the state of the target. It perfectly avoids the motor engine disturbance of the vehicle target when the sensors are in the process of delivering data and the radio communication quality can be greatly enhanced.

### 3.1. TPLE Algorithm for Link Quality Estimation

As we mentioned before, the extra complexity of on-road traffic context lies in the unpredictable and sudden appearance of a vehicle target. Vehicle engines cause great disturbance to wireless links when they are close to them.

According to our previous work in the on-road environment, the radio communication quality of a sensor node will decline when vehicle engines appear around a sensor. So an intelligent on-road data delivery scheme should be established for a radio communication task by reducing the disturbances of traffic motor targets.

Assume that a motor engine’s magnetic field is like a disk having radius of R. The motion model of a nearby vehicle can be described as follows:

(1) $$x_{k+1}=F_{k}x_{k}+G_{k}u_{k}+w_{k}$$

The measurement model of a sensor node is as follows:

(2) $$z_{k}=H_{k}x_{k}+v_{k}$$

In these two equations, $x_{k}$ is the state vector at time instant k, $F_{k}$ is the targets matrix of state transition, $G_{k}$ is the matrix of input noise, $u_{k}$ is the dynamic noise, $w_{k}$ is the process noise, $Z_{k}$ is the measurement vector of sensor node, $H_{k}$ is the measurement matrix, $v_{k}$ is the measurement noise.

By adopting target tracking methods [18,19,20], such as Kalman filter [21], the target states such as speed and position can be estimated. According to the radius R of the influential scope, the time instant when a target leaves the influential scope can be predicted. When a target’s track is estimated by a sensor node, the sensor node can reschedule the transmission task and improve its quality.

Based on this idea, we propose a target-prediction-based link quality estimation mechanism, (TPLE). In TPLE mechanism, the downstream nodes can be informed appearance of an incoming vehicle target and dynamically calculate the link quality at local. By this mechanism, the radio communication quality can be greatly enhanced.

### 3.2. Reliable Data Delivery Scheme

In this part, we describe the TPLE-based reliable data delivery scheme. First, we introduce some scenarios that are very common in an on-road traffic monitoring environment. Figure 2 shows typical on-road monitoring settings. Several sensor nodes are deployed along a road, the sensor nodes are fixed in position, the distance between two neighbor nodes is d(For the simplicity consideration, here we suppose the distance between neighbor nodes are equal in one system) [22]. Also, we suppose that the vehicle target is in a state of uniform rectilinear motion. We suppose the sensor nodes are synchronized with their local time in the system.

**Figure 3:**
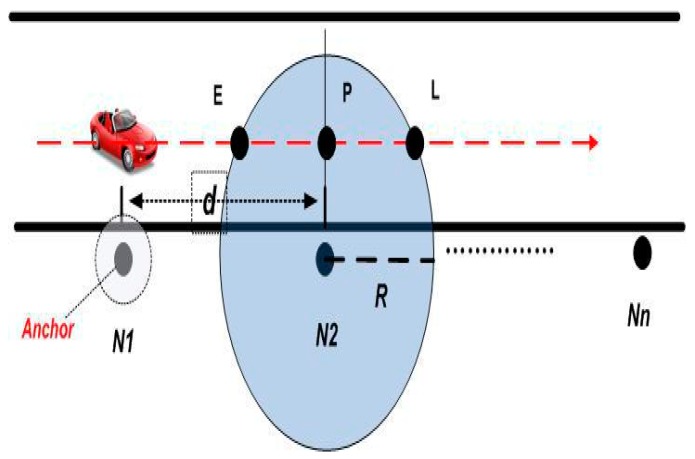
Monitoring settings.

Here we introduce the concept of an Anchor. We define an Anchor to be the first node in a monitoring system that notices the incoming target. According to [23], a sensor node can accurately report the time instant of a passing vehicle. When the vehicle target is monitored by a sensor node $N_{m}$, this sensor node can produce a message of feature vector $I_{m}$, where $I_{m}=\{t_{m},s_{m}\}$. Here, $t_{m}$ is the time instant that the target passing a sensor node $N_{m}$, $s_{m}$ is the signal strength of the monitoring target when the target reaches a sensor node $N_{m}$. In Figure 3, $N_{1}$ is supposed to be the Anchor. When a vehicle target is identified by an Anchor, a message of feature vector $I_{1}$ will be broadcast to its relevant neighbors $N_{2}$ in this figure.

As the data transmission process may possibly fail to accomplish the task, in a real system, a simple ACK-based retransmission mechanism, for such acknowledgement provides an Active Message in TinyOS [24] that can be added to an Anchor to ensure that this packet transmission process has been successfully performed.

As to the relevant neighbor, $N_{2}$ in Figure 3, it will be informed the feature vector of an incoming target from upstream $N_{1}$. Meanwhile, in a short time, $N_{2}$ produces its own feature vector about that target $I_{2}$, $I_{2}=\{t_{2},s_{2}\}$. With the combination of $I_{1}$ and $I_{2}$, $N_{2}$ can obtain an estimation of target speed locally:

(3) $$\bar{v}=\frac{d}{\left|t_{2}-t_{1}\right|}$$

where *d* is the distance between sensor $N_{1}$ and $N_{2}$; $t_{1}$, $t_{2}$ is the time instant when the target reaches the sensor $N_{1}$, $N_{2}$ respectively, the value of them is derived from feature vector $I_{1}$ and $I_{2}$. With the estimated target speed $\bar{v}$, $N_{2}$ can predict the target track. The leaving time of the coming target can be estimated as:

(4) $$\bar{t_{l}}=t_{2}+\frac{d_{PL}}{\bar{v}}$$

where $d_{PL}$ is the distance between point P and point L. Because it is technically difficult to calculate the value of $d_{PL}$, we replace $d_{PL}$ with R in (1), and get $\bar{{t^{\prime }}_{l}}$. As R > $d_{PL}$, so $\bar{{t^{\prime }}_{l}}$ ≥ $\bar{t_{l}}$. Besides simplicity, another advantage of this replacement is that the increased $\bar{t_{l}}$ assures the package transmission which will be scheduled upon vehicle departure. The tradeoff of this replacement is the tiny increment in package delay. After this calculation of $t_{l}$, $t_{l}$ can schedule its radio transmission tasks locally.

As we define the Anchor to be the first node that discovers the incoming target. In some cases, the physically first node which is supposed to discover the vehicle target may miss the target and fail to report to relevant nodes. In such a case, the first discovery of that vehicle serves as the Anchor and finishes the report task.

**Figure 4:**
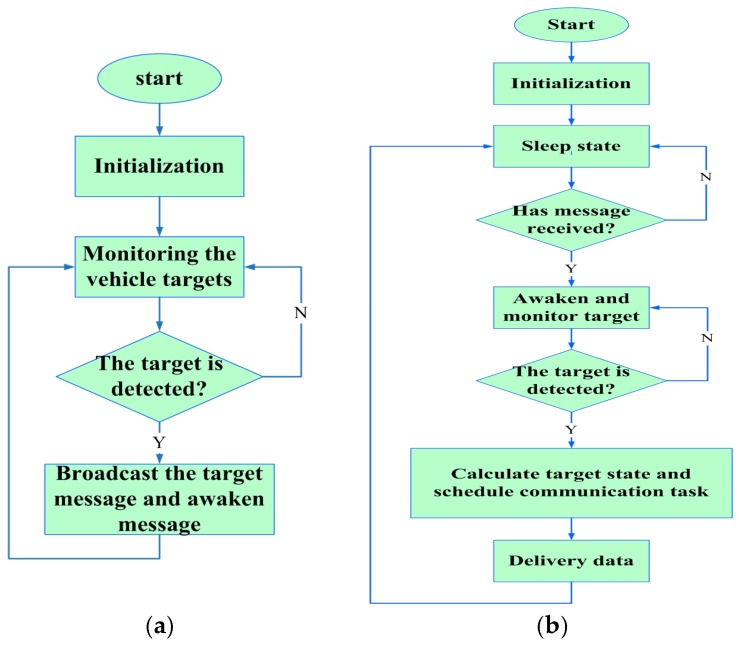
The diagram chart of TPLE, where (**a**) represents the flow diagram of the Anchor; (**b**) represents the flow diagram of sensor nodes.

As all the sensor nodes in our system are homogenous, it is not difficult to implement the aforementioned mechanisms in a sensor node. Figure 4 indicates a diagram chart of our proposed TPLE algorithm. 

## 4. Verification and Experiment

In traditional wireless communication a periodic sleep-wake mechanism for sensor nodes has been designed and employed in order to save energy better, in which every node in the sleep mode periodically wakes up to communicate with its neighbor nodes [25]. Once the communication is over the node goes in to sleep mode again until the next frame begins. However, in traffic monitoring circumstances, the sensor nodes need to monitor targets frequently and communicate with their neighbors during active time. Besides, a node cannot receive messages during sleep mode, so, the messages targeted for such a node may be lost. In such a situation, the traditional duty cycle mechanism can neither save energy effectively nor obviously improve transmission quality of the WSN. The TPLE algorithm we proposed can predict the target status and then the active opportunity of sensor nodes can be estimated. Our algorithm does not need to monitor targets frequently, especially if the data delivery is in an environment without motor engine disturbance. Therefore, our TPLE algorithm can save energy of the WSN effectively and better improve the quality of data delivery. In this part, we conducted both simulations and on-road experiments to verify our proposed scheme.

### 4.1. Simulations

We conduct computer simulations to verify our TPLE data delivery scheme. In our simulations, a sensor node is supposed to report its measurement when a vehicle target passes from its side.

(1) Baseline algorithms and simulation settings

We use two algorithms as comparison baselines, a simple acknowledgement-based single hop delivery provided by CC1000 radio stack (CRS) of Active Message in TinyOS [24] and EWMA algorithm adopted in [26]. The maximal retransmission number in the MRS algorithm is set to be 3. The length of temporal statistical window in EWMA is set to be 10 s. The EWMA algorithm sends a probing message every 0.5 s. In EWMA, a link is supposed to be good if the statistical package delivery rate is higher than 0.7.

The data delivery rate, average package delay, energy consumption, and expected package retransmission lead to performance evaluations.

As vehicles are the largest disruption to link quality, we adopt different traffic densities (low, medium, high) to evaluate the performances. In low traffic density, we generate 5 vehicle appearances every minute; in the medium density, 10 vehicles are generated every minute; and in the high density, 20 vehicles are generated every minute. For each traffic density, we conducted 20 groups of simulation.

The simulation settings are listed in Table 1. In Table 1, according to a large amount of on-road collected data, we set the radius of sensor nodes radio influence to 4 m, which means a vehicle in this range will reduce the sensor nodes’ wireless communication quality. Also, we assigned 0.80, which is a relatively ideal radio communication quality in outdoor non-vehicle-appearance environment. The minimal distance between two vehicles is set to be 5 m, which is a normal setting in the case of no traffic jam. Moreover, our proposed TPLE mechanism is applicable in this assumption. The Baud rate of a sensor node is assigned to be 19.2 kbps, which is a common rate in CC1000 radio communication modules. The packet size has been fixed to 28 bytes, which is the standard packet size in TinyOS. We use the Baud Rate and Packet size to calculate packet delay between sender and receiver; we ignore the other factors like transmission delay and software delay.

(2) Data Delivery Rate

**Figure 5:**
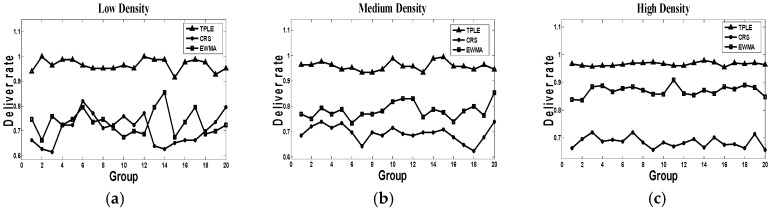
Data delivery rate simulation, where (**a**) represents the data delivery rate in low traffic density; (**b**) represents the data delivery rate in medium traffic density; (**c**) represents the data delivery in high traffic density. The representation of horizontal coordinates is the groups of simulation, the representation of vertical coordinates is the data delivery rate.

The data delivery rate is an important criterion for link quality. Figure 5 indicates the data delivery results in the simulation. According to this figure, the data delivery rate of our proposed TPLE scheme is higher than 94% in different traffic density, which is very ideal. The delivery rate of CRS algorithm in all three traffic scenarios remains consistent to 70% almost. The reason is that the CRS algorithm does not take any link quality into consideration, the ACK mechanism is the only solution for high data delivery rates. As to the EWMA algorithm, although it takes the link state into consideration, its link state estimation accurately reflects the real link status. In the high traffic density scenario, the performance of EWMA is mostly observed as being unstable. The reason for this phenomenon is that dense traffic introduces extra-high unpredictability factors for the link state estimation parts of EWMA.

To summarize, our proposed TPLE scheme provides a stable and high data delivery rate for on-road sensors, it deals well with the different traffic scenarios.

(3) Average packet delivery delay

**Figure 6:**
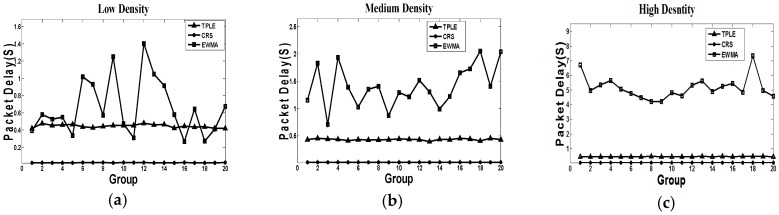
Packet delay simulation, where (**a**) represents the packet delay in low traffic density; (**b**) represents the packet delay in medium traffic density; (**c**) represents the packet delay in high traffic density. The representation of horizontal coordinates is the groups of simulation, the representation of vertical coordinates is the packet delay.

Figure 6 indicates the packet delay in simulation. EWMA algorithm has the largest average packet delay; the reason is that the link estimation in the EWMA algorithm enables wireless transmission when the statistical link quality is better than the threshold. The target-related information transmission experiences an unavoidable delay because of the statistical link quality estimation mechanism. The CRS algorithm has the smallest delivery delay because it starts packet transmission once the target is detected. The cost of low packet delay is a relatively high retransmission number and low packet delivery rate. We will demonstrate it later. Our proposed TPLE has a relatively high delivery delay, because it schedules transmission task when the target leaves, not like the immediate transmission in the CRS algorithm.

(4) Average packet retransmission number

**Figure 7:**
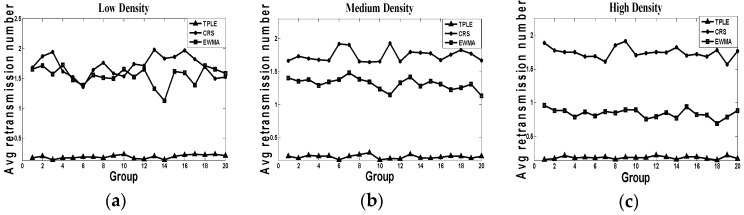
Average retransmission number, where (**a**) represents the packet retransmission number in low traffic density; (**b**) represents the packet retransmission number in medium traffic density; (**c**) represents the packet retransmission number in high traffic density. The representation of horizontal coordinates is the groups of simulation, the representation of vertical coordinates is the average packet retransmission number.

Figure 7 indicates the average packet retransmission number in the simulation. From this figure, we can see along with the change in traffic density, the packet retransmission number of CRS and TPLE algorithms keep stable. TPLE has the lowest packet retransmission number while the CRS algorithm has the largest retransmission number. The average retransmission number of the EWMA algorithm decreases as the traffic density increases. The reason for this phenomenon is that more traffic results in the link quality estimation of EWMA becoming more accurate and the transmission quality is improved. Compared with two baseline algorithms, TPLE has the least retransmission because it schedules the radio communication tasks when it has a relatively good link quality.

(5) Energy consumption

**Figure 8:**
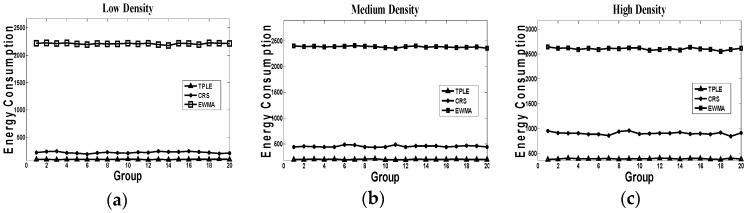
Energy cost simulation, where (**a**) represents the energy consumption in low traffic density; (**b**) represents the energy consumption in medium traffic density; (**c**) represents the energy consumption in high traffic density. The representation of horizontal coordinates is the groups of simulation, the representation of vertical coordinates is the energy consumption.

As radio communication accounts for the greatest energy cost of a sensor node, we use total packet number to evaluate the energy consumption. In our algorithm, we use the CC1000 as a communication module which with very low current consumption, its unit energy consumption is the change of energy in unit time at a certain power. In the three algorithms (EWMA, CRS, and TPLE), we define the total packet number, the transmission power, and the transmission time as the reference standard for energy consumption. The working power of the CC1000 can be set before the experiment. So, we can evaluate the energy consumption according to the total packet number of the sensors. From Figure 8, we can see the EWMA algorithm definitely has the largest energy consumption because of the periodical link probing messages. As TPLE adopts the most effective link estimation mechanism, it consumes the least energy.

### 4.2. On-Road Verification Experiments

**Figure 9:**
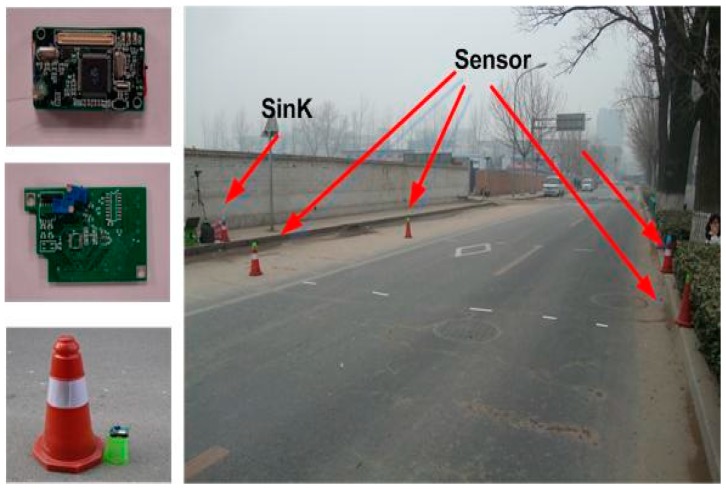
On-Road experiment scenario.

We also conducted real experiments to verify our proposed data delivery scheme. The experiments can be seen in Figure 9. Based on the platform EasiTia [23], we implemented the TPLE algorithms in sensor nodes.

Then the sensor nodes were deployed alongside a road to detect and report vehicle messages. Figure 8 indicates our on-road experiment settings. We used four nodes along the road-side to perform data collection tasks, two nodes on each side of the road. A sensor node is supposed to send its measurement about a vehicle to a sink node.

We conducted three groups of experiments, each experiment lasted 10 min, we use packet delay, packet delivery rate, and retransmission numbers to evaluate our experiment result. Table 2 indicates results of our on-road experiments. We compared the three algorithms (EWMA, CRS, and TPLE algorithms) in delivery rate, packet delay, and retransmission number. In Table 2, Group 1, Group 2, and Group 3 represent the EWMA algorithm, the CRS algorithm, and the TPLE algorithm respectively. Each experiment of three algorithms is the same, and the result values of delivery rate, packet delay, and retransmission number are the average values of all the experiments.

From this table we can see that, the on-road data delivery rate of TPLE is not as good as the results in simulations. The reason of this phenomenon may result from the inaccurate definition of the influential scope of the sensors’ radio link. However, we may see that the packet delivery delay and average retransmission number is ideal as indicated by our simulation.

On the basis of both simulations and on-road experiments, we verified our proposed TPLE data delivery scheme. We demonstrated that our proposed scheme performed relatively better data delivery with less cost for a small packet delivery. The delay is acceptable in an on-road monitoring environment.

## 5. Conclusions

In this paper, we propose a TPLE mechanism to solve the problem of low link quality caused by motor engines in the on-road traffic monitoring environment. TPLE estimates link quality by dynamically updating target motion status and scheduling radio communication tasks when there is no motor engine disturbance. Simulation and on-road experiments demonstrate the good communication quality of TPLE in on-road environments.

## Figures and Tables

**Table 1:** Simulation Settings.

Parameter	Value	Description
Dn2n-Dn2n	6 m	Distance between neighbor nodes
V	5 m/s–15 m/s	Vehicle speed
M_ax_D_v2v_m_ax_D_v2v_	5 m	Minimal distance between two vehicles
R	4 m	Radius of a nodes magnetic field
F1-F1	0.8	Packet delivery rate without vehicle in influential scope
Pf-Pf	0.25	Packet delivery rate with vehicle in influential scope
Baud rate	19.2 kbps	Radio rate
Packet size	28 bytes	Radio packet size 0

**Table 2:** On-road experiment results.

	Delivery Rate	Packet Delay	Retransmission Number
Group 1	82.54%	1.08s	0.71
Group 2	85.87%	0.84s	0.73
Group 3	94.11%	0.81s	0.61
